# Robotic-assisted colorectal surgery in colorectal cancer management: a narrative review of clinical efficacy and multidisciplinary integration

**DOI:** 10.3389/fonc.2025.1502014

**Published:** 2025-04-07

**Authors:** Engeng Chen, Li Chen, Wei Zhang

**Affiliations:** Department of Colorectal Surgery, Sir Run Run Shaw Hospital of Zhejiang University School of Medicine, Hangzhou, China

**Keywords:** robotic-assisted colorectal surgery, multidisciplinary treatment strategies, colorectal cancer, narrative review, clinical efficacy

## Abstract

Colorectal cancer (CRC) remains a formidable global health challenge, ranking among the most prevalent malignancies and a principal contributor to cancer-associated mortality. While traditional open surgery has historically been the cornerstone of CRC treatment, the advent of minimally invasive techniques, particularly robotic-assisted colorectal surgery (RACS), has garnered significant momentum owing to technological advancements in the field. Robotic platforms, exemplified by the da Vinci Surgical System, offer superior three-dimensional visualization, enhanced dexterity, and heightened precision, yielding improved perioperative outcomes, particularly in anatomically intricate regions such as the pelvis. This review provides a critical appraisal of the current landscape of RACS, emphasizing its superiority over conventional open and laparoscopic approaches. The increased control and precision afforded by robotic surgery have been shown to optimize outcomes in complex procedures such as total mesorectal excision, with evidence indicating reduced intraoperative blood loss, shortened hospital stays, and improved functional recovery. Nonetheless, challenges persist, including absence of haptic feedback, prohibitive costs, and steep learning curve associated with robotic systems. Despite these limitations, RACS has demonstrated considerable promise in sphincter-preserving and function-preserving procedures, ultimately enhancing postoperative quality of life. Beyond the surgical field, this review also investigates the integration of robotic surgery within multidisciplinary treatment strategies for CRC, particularly in the context of locally advanced rectal cancer. The combination of robotic techniques with total neoadjuvant therapy and immunotherapy—especially in tumors characterized by mismatch repair deficiency or high microsatellite instability has shown notable clinical efficacy. Furthermore, emerging personalized therapeutic approaches, including immunotherapies and targeted chemotherapeutic agents, emphasize the transformative potential of RACS in delivering superior oncologic outcomes. Looking towards the future, innovations in robotic platforms, including intraoperative imaging, artificial intelligence, and augmented reality, herald new possibilities for further enhancing the precision and efficacy of colorectal surgeries. The standardization of RACS protocols, alongside ongoing training and robust clinical research, will be critical to fully realizing the benefits of these advancements across diverse clinical settings. By incorporating cutting-edge technologies and personalized treatment methods, robotic-assisted surgery is prepared to become a cornerstone in future of CRC management, with the potential to significantly improve both survival outcomes and patient quality of life.

## 1 Introduction

Colorectal cancer (CRC) remains a significant global health burden, ranking among the most prevalent malignancies and contributing to a substantial number of cancer-related deaths (1). According to the International Agency for Research on Cancer, CRC accounts for more than 1.9 million new cases annually, with an estimated 900,000 fatalities, representing 9.3% of all cancer-associated mortalities (2). Traditionally, open colorectal resection has been the cornerstone of surgical intervention for CRC (3). Although effective in achieving tumor resection and improving survival outcomes, open surgery is accompanied by notable limitations, including extensive surgical trauma, increased complication rates, and prolonged postoperative recovery (4).

Over the past few decades, advances in medical technology have catalyzed the widespread adoption of minimally invasive surgery (MIS) (5). Laparoscopic surgery, as the foundation of MIS, has gained considerable traction in the management of CRC (6). Compared to conventional open approaches, laparoscopic techniques offer several distinct advantages, such as reduced intraoperative trauma, diminished postoperative pain, accelerated recovery, and improved cosmetic outcomes (7). However, despite these benefits, laparoscopic surgery has inherent drawbacks, including a two-dimensional (2D) visual field, restricted instrument dexterity, and a steep learning curve, all of which can compromise surgical accuracy and hinder its broader implementation in clinical practice (8, 9).

**Figure 1:**
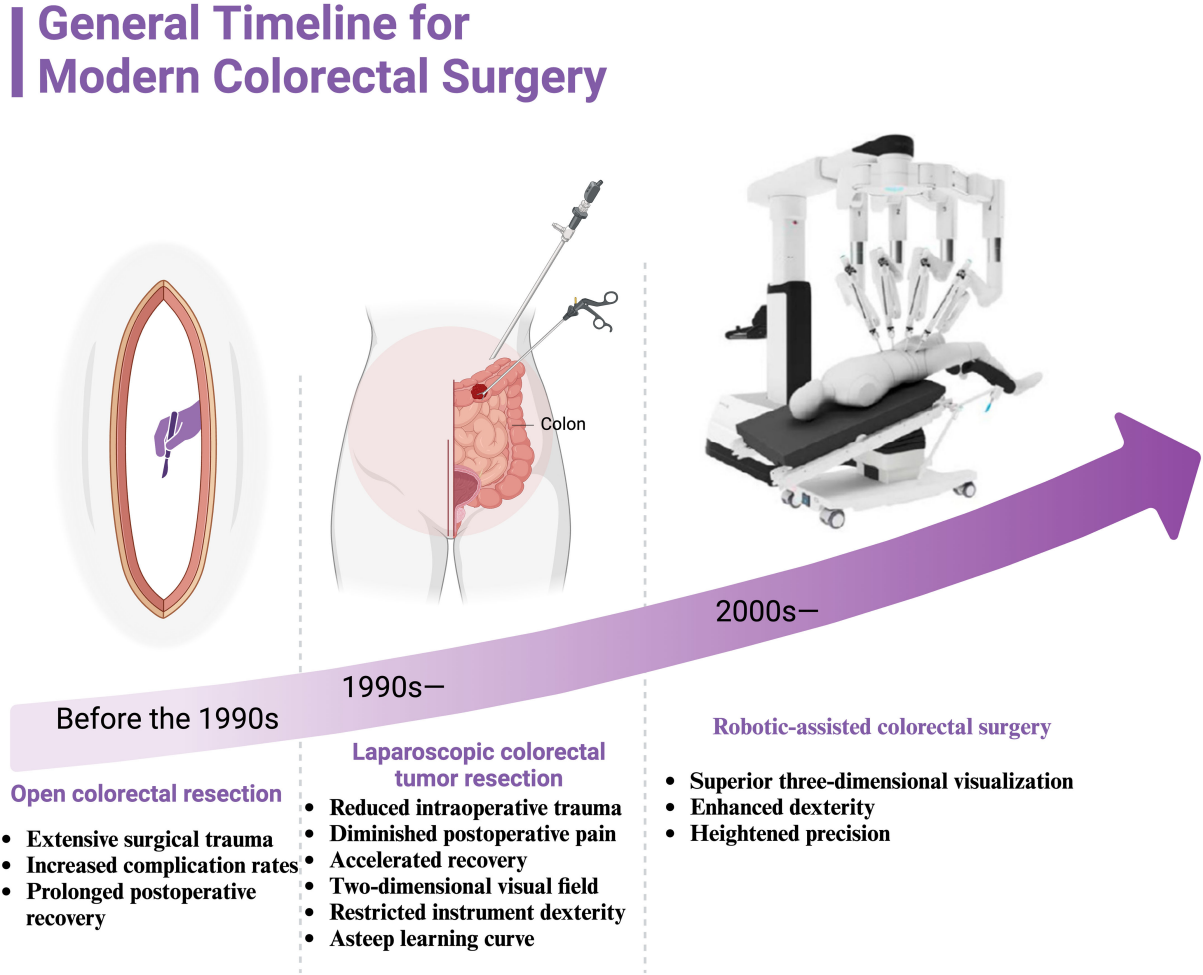
General timeline for modern colorectal surgery. It illustrates the progression of surgical techniques for colorectal cancer treatment from the pre-1990s era of open colorectal resection, through the development of laparoscopic colorectal tumor resection in the 1990s, to the introduction of robotic-assisted colorectal surgery in the 2000s. Before the 1990s, open colorectal resection was the standard procedure, characterized by extensive surgical trauma, higher complication rates, and prolonged postoperative recovery. In the 1990s, laparoscopic colorectal tumor resection emerged, offering advantages such as reduced intraoperative trauma, diminished postoperative pain, and accelerated recovery. However, challenges included the limitations of a two-dimensional visual field, restricted instrument dexterity, and a steep learning curve for surgeons. Since the 2000s, robotic-assisted colorectal surgery has provided superior three-dimensional visualization, enhanced dexterity, and heightened precision, marking a significant advancement in the field of minimally invasive surgery.

To address these limitations, robot-assisted surgical systems have emerged as a transformative innovation. The da Vinci Surgical System, for instance, has revolutionized the surgical landscape by introducing robot-assisted surgery (RAS) into clinical practice, offering enhanced capabilities such as high-definition three-dimensional (3D) visualization, instruments with seven degrees of freedom, and tremor suppression, significantly augmenting surgical precision (10). In the context of CRC surgery, RAS holds promise for improving lymph node dissection quality, reducing perioperative complications, and optimizing functional outcomes, particularly in sphincter-preserving procedures (11). (**Figure 1**).

As a narrative review, our objective is to synthesize the current body of evidence on robotic-assisted colorectal surgery and its integration into multidisciplinary treatment strategies for CRC. This format allows for a comprehensive examination of both established and emerging data, enabling us to provide a critical overview without restricting the scope to predefined methodological criteria, as might be required in a systematic review. By presenting the literature in this narrative format, we aim to highlight the nuanced clinical insights, evolving technologies, and multidisciplinary considerations that shape the use of robotics in CRC management.

## 2 Robotic-assisted colorectal surgery

### 2.1 Current status and trends

The landscape of robotic-assisted colorectal surgery has witnessed remarkable growth in recent years, primarily fueled by continuous innovations in robotic technology (12). These advancements have significantly enhanced the precision and efficacy of surgical interventions, especially in intricate or technically demanding cases (13). Central to this paradigm shift is the advent of high-definition, three-dimensional imaging and fully articulating robotic instruments, which afford surgeons unprecedented dexterity and spatial awareness (14). These tools enable the execution of complex procedures with heightened control, mitigating the limitations inherent to traditional open or laparoscopic techniques.

Recent studies emphasize the increasing utilization of robotic platforms in colorectal surgeries that have historically posed considerable challenges (15–17). Notably, robotic systems are now routinely employed for lateral lymph node dissections and multi-visceral resections involving adjacent organs, both critical for addressing locally advanced malignancies (18–21). This evolution mirrors growing confidence within the surgical community regarding the capabilities of robotic systems in managing complex colorectal pathologies (22). Such advancements are contributing to the broader adoption of robotic-assisted surgery across diverse colorectal cancer cases, particularly within specialized, high-volume centers where the requisite infrastructure and expertise are readily available (23).

### 2.2 Comparison with conventional methods

Robotic-assisted colorectal surgery offers a range of advantages over conventional open and laparoscopic approaches, while also presenting unique challenges (24, 25). Laparoscopic surgery, long regarded as the cornerstone of minimally invasive colorectal surgery, is limited by two-dimensional imaging and a constrained range of motion due to rigid, non-articulating instruments (26, 27). These limitations can impede precise dissection, particularly within confined anatomical spaces such as the pelvis (28). Open surgery, while providing direct tactile feedback and a broader operative field, is associated with increased morbidity, including greater postoperative pain, prolonged hospitalization, and slower recovery (29).

Robotic systems bridge these gaps by offering the enhanced dexterity of wristed instruments and superior visualization through high-definition, three-dimensional imaging (30). This capability facilitates more refined dissections in anatomically complex regions, such as during total mesorectal excision (TME) for rectal cancer (31, 32). Comparative studies demonstrate that robotic-assisted surgery can lead to reduced intraoperative blood loss, lower rates of conversion to open surgery, and shorter hospital stays relative to both laparoscopic and open procedures (33). Nonetheless, robotic surgery is not without drawbacks, including extended operative times and higher upfront costs related to the acquisition and maintenance of robotic platforms (34). While long-term oncological outcomes remain comparable between robotic and conventional methods, further research is necessary to determine whether robotic systems offer superior long-term survival or recurrence rates for patients with colorectal cancer (35, 36).

### 2.3 Challenges in robotic surgery

**Figure 2:**
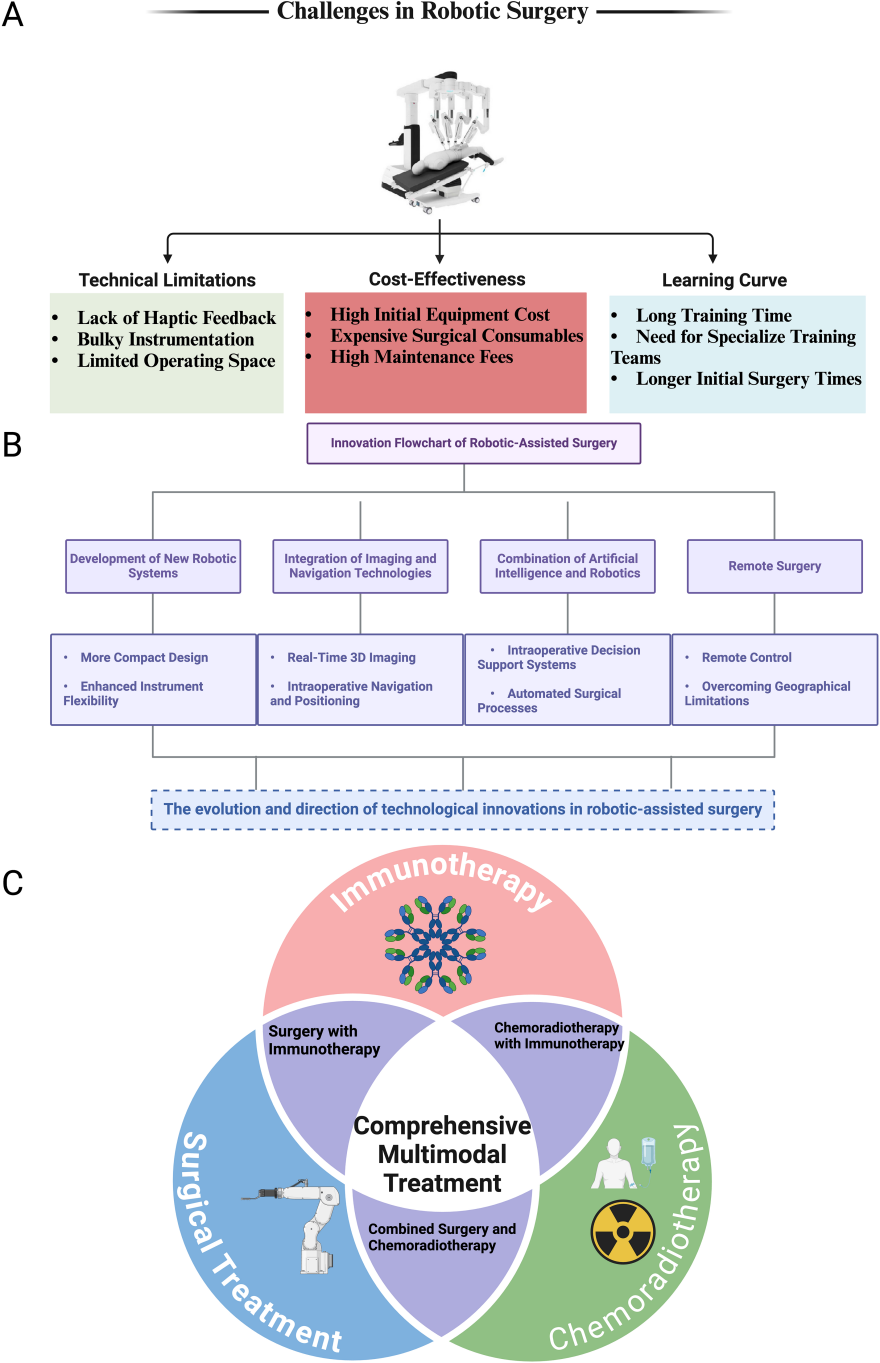
Challenges, innovations, and multimodal roles of robotic-assisted surgery in colorectal cancer treatment. **(A)** Challenges in Robotic Surgery: This panel illustrates the three primary challenges associated with robotic-assisted surgery: Technical Limitations: Including the lack of haptic feedback, bulky instrumentation, and limited operating space. Cost-Effectiveness: Highlighting the high initial equipment costs, expensive surgical consumables, and high maintenance fees. Learning Curve: Emphasizing the long training times, the need for specialized training teams, and longer initial surgery times for surgeons adopting this technology. **(B)** Innovation Flowchart of Robotic-Assisted Surgery: This panel presents the technological advancements driving the evolution of robotic surgery: Development of New Robotic Systems: Focused on creating more compact designs and enhancing instrument flexibility. Integration of Imaging and Navigation Technologies: Including real-time 3D imaging and intraoperative navigation and positioning. Combination of Artificial Intelligence and Robotics: Introducing intraoperative decision support systems and automated surgical processes. Remote Surgery: Enabling remote control and overcoming geographical limitations for surgical interventions. **(C)** Comprehensive Multimodal Treatment: A Venn diagram representing the role of robotic-assisted surgery in the context of multimodal treatment strategies for colorectal cancer. The three intersecting circles represent: Surgical Treatment, Chemoradiotherapy, and Immunotherapy. The overlap between these treatment modalities highlights the integration of robotic surgery in Surgery with Immunotherapy, Combined Surgery and Chemoradiotherapy. The center of the diagram emphasizes the importance of Comprehensive Multimodal Treatment in optimizing patient outcomes for colorectal cancer treatment.

Despite the clear advantages of robotic-assisted colorectal surgery, several technical and operational challenges persist. A major limitation is the absence of haptic feedback, which is integral for surgeons to gauge tissue tension and resistance during dissection (37). The lack of tactile sensation can impair the precision of critical maneuvers, particularly when working near delicate structures. Additionally, the considerable size of robotic systems and the complexity of docking procedures can prolong operative times, particularly in centers where robotic surgery has not yet become routine (38). (**Figure 2A**).

The high cost of robotic platforms, both in terms of initial investment and ongoing maintenance, also represents a significant barrier, especially for smaller hospitals or those in resource-limited settings (39, 40). The cost-effectiveness of robotic surgery remains a subject of ongoing debate, particularly in scenarios where laparoscopic techniques can achieve comparable outcomes at a fraction of the cost (41). Furthermore, the learning curve associated with robotic-assisted surgery is steep (42). Mastery of these systems requires specialized training and substantial experience, which can delay widespread adoption and potentially lead to variability in outcomes during the initial phase of implementation. Addressing these challenges through enhanced system designs, cost-reduction initiatives, and the development of more comprehensive training programs will be critical to improving the accessibility and efficacy of robotic-assisted colorectal surgery.

## 3 Multidisciplinary management of locally advanced rectal cancer

### 3.1 Total Neoadjuvant Therapy

Total Neoadjuvant Therapy (TNT) has emerged as a pivotal strategy in the management of locally advanced rectal cancer (LARC), integrating both preoperative chemoradiotherapy and systemic chemotherapy prior to surgical resection (43–45). The primary objective of TNT is to optimize oncological outcomes by enhancing tumor downstaging, increasing the rates of pathological complete response (pCR), and improving both disease-free survival (DFS) and overall survival (OS) (46). In contrast to traditional treatment methods, where adjuvant chemotherapy follows surgical intervention, TNT prioritizes systemic chemotherapy in conjunction with neoadjuvant radiotherapy before surgery (47). This approach increases the likelihood of achieving negative surgical margins and addresses micrometastatic disease early in the treatment course.

Administering systemic chemotherapy prior to surgery confers multiple advantages, particularly in controlling micrometastatic disease at an earlier stage, which may reduce the risk of distant metastasis (48). Moreover, completing chemotherapy preoperatively ensures that patients—especially those at heightened risk of postoperative complications or those less likely to tolerate adjuvant therapy due to delayed recovery—receive the full therapeutic benefit. Clinical trials have consistently demonstrated TNT’s efficacy in increasing pCR rates, a surrogate marker strongly correlated with improved long-term outcomes, thereby cementing its clinical value (49, 50).

### 3.2 Role of immunotherapy

In the evolving treatment landscape of rectal cancer, the integration of immunotherapy, particularly immune checkpoint inhibitors (ICIs), represents a significant advancement, especially for tumors characterized by mismatch repair deficiency (dMMR) or high microsatellite instability (MSI-H) (51). These tumors, known for their high mutational burden, exhibit enhanced immunogenicity, making them highly susceptible to T-cell-mediated cytotoxicity via immunotherapy (52, 53). For patients with dMMR/MSI-H rectal cancer, ICIs have demonstrated remarkable clinical efficacy, often yielding profound radiologic and clinical responses, with some cases obviating the need for conventional chemoradiotherapy or surgery (54).

Agents such as pembrolizumab and nivolumab have shown high response rates in dMMR/MSI-H patients, enabling treatment de-escalation and significantly improving quality of life by sparing patients from the morbidity associated with traditional, more aggressive treatments (51). These responses are supported by the immune system’s enhanced ability to recognize and eliminate cancer cells in dMMR/MSI-H tumors, driven by their inherent genomic instability. This paradigm shift has fundamentally altered the therapeutic approach for this subset of patients.

Nonetheless, successful immunotherapy hinges on precise patient selection. dMMR or MSI-H status must be confirmed through comprehensive genomic profiling, as patients without these molecular features—namely those with microsatellite-stable (MSS) tumors—derive minimal benefit from ICIs (55). Ongoing research is exploring combinatorial strategies, such as pairing immunotherapy with chemotherapy or radiotherapy in MSS tumors, with the aim of broadening the applicability of ICIs and improving outcomes for a wider patient cohort (56).

### 3.3 Necessity of personalized therapeutic approaches

Given the rapid advancements in rectal cancer treatment, the imperative for individualized therapeutic strategies has never been more critical (57). While both TNT and immunotherapy represent significant progress, their optimal implementation demands careful tailoring to each patient’s unique clinical and molecular characteristics. Personalizing treatment necessitates consideration of several factors, including tumor genetics, patient comorbidities, and the tumor’s responsiveness to initial therapeutic interventions (58).

In elderly or frail patients, for instance, the intensity of TNT may require modification to reduce treatment-related toxicity while maintaining therapeutic efficacy (59). Conversely, younger or more resilient patients may be candidates for aggressive multimodal approaches aimed at maximizing oncological control (60). The role of multidisciplinary teams—comprising surgical oncologists, medical oncologists, radiation oncologists, and genetic counselors—is indispensable in crafting these individualized treatment regimens, which strive to balance optimal oncological outcomes with quality-of-life considerations.

Advancements in surgical technology, particularly robotic-assisted techniques, further enhance the capacity for personalized care. This is especially pertinent for patients with low rectal tumors, where function-preserving procedures are a priority (61, 62). The integration of these technologies with personalized preoperative and postoperative strategies enables improved functional outcomes while maintaining rigorous cancer control.

## 4 Innovations and standardization in robot-assisted colorectal surgery

### 4.1 Technological advancements

Recent advances in robot-assisted colorectal surgery (RACS) have marked the dawn of a new era in surgical precision and patient safety, with next-generation robotic platforms like the da Vinci Xi system at the forefront of these developments (63). These systems offer superior dexterity, enhanced stability, and refined tactile feedback, features that are particularly vital for executing intricate colorectal procedures in anatomically restrictive regions such as the pelvis (64). The expanded range of motion and more intuitive user interface afforded by these platforms have not only improved surgical precision but also enabled surgeons to navigate challenging anatomical planes with greater ease, thereby reducing the technical complexity inherent in operations such as low anterior resection and TME.

Among the most transformative innovations is the integration of intraoperative fluorescence imaging, a method that allows real-time visualization of tissue perfusion, enhancing the surgeon’s ability to delineate resection margins with unparalleled accuracy (65, 66). This technology is pivotal in reducing the risk of anastomotic leaks—one of the most feared complications in colorectal surgery. Additionally, the advent of 3D imaging and augmented reality (AR)-based navigation systems has significantly augmented the surgeon’s capacity to visualize and manipulate within the operative field (67), particularly in the dense and complex anatomy of the pelvis. These tools have refined the precision of resection planes, minimized collateral damage to adjacent tissues, and improved oncological clearance, especially in minimally invasive surgical contexts. (**Figure 2B**).

### 4.2 Standardization efforts

With the increasing adoption of RACS across diverse clinical settings, variability in surgical techniques and patient outcomes remains a pressing concern. In response, considerable efforts have been made to standardize the practice of robotic-assisted colorectal surgery (9, 68). Professional organizations and surgical societies have developed comprehensive guidelines designed to harmonize key aspects of robotic surgery, including preoperative planning, patient selection, and intraoperative procedures (69). These guidelines seek to ensure the consistent application of critical steps—such as optimal patient positioning, standardized port placement, and robotic docking techniques—across institutions, thereby enhancing the reproducibility and quality of surgical outcomes.

In tandem with these procedural guidelines, the establishment of rigorous training and accreditation frameworks has further bolstered the standardization of RACS. Simulation-based training programs offer a safe and controlled environment where surgeons can refine their technical skills before transitioning to live surgery (70). Competency evaluations, coupled with proctored surgeries, form an integral part of certification processes, ensuring that only those surgeons who have demonstrated both technical proficiency and adherence to standardized protocols are entrusted with performing robotic procedures independently. Furthermore, continued medical education (CME) requirements ensure that surgeons remain abreast of the latest technological innovations and procedural refinements, fostering a culture of continuous improvement in surgical practice (71).

### 4.3 Function-preserving techniques

In colorectal surgery, particularly in the management of malignancies located in the lower rectum, there has been a growing emphasis on techniques that preserve function, aiming to improve outcomes not only in terms of oncological control but also in the quality of life for patients (72). One notable advancement in this regard is the development of sphincter-preserving procedures, such as intersphincteric resection (ISR) (73). ISR allows for the excision of rectal tumors while preserving the sphincter complex, thereby avoiding the need for permanent colostomies and significantly enhancing patient quality of life. The enhanced precision provided by robotic systems is particularly beneficial in these cases, enabling the meticulous dissection required to protect critical structures such as the sphincter complex and pelvic nerves.

Robotic platforms have also revolutionized the performance of TME, which remains the cornerstone of rectal cancer surgery (74). TME demands exacting dissection to maintain the integrity of the mesorectal fascia while ensuring comprehensive oncological resection (75). The fine motor control and superior visualization offered by robotic systems minimize the risk of inadvertent nerve damage, thereby preserving essential pelvic functions such as urinary and sexual function (76). This heightened precision is directly correlated with improved postoperative functional outcomes, translating into both enhanced cancer cure rates and better overall quality of life for patients following surgery.

Therefore, the integration of state-of-the-art robotic technologies into colorectal surgery, alongside concerted efforts to standardize surgical protocols and embrace function-preserving techniques, heralds a paradigm shift in the treatment of colorectal diseases. These advancements not only offer superior oncological outcomes but also foster improved functional recovery, addressing critical issues related to postoperative morbidity and long-term quality of life.

## 5 Minimally invasive surgery: curative and functional outcomes

### 5.1 Oncological outcomes

MIS has become integral to the treatment of colorectal cancer, with robotic-assisted techniques representing a considerable leap forward in surgical innovation. The primary oncological benchmarks used to assess the efficacy of these methods include survival rates and local recurrence rates, both of which are pivotal in determining long-term disease control (77).

In terms of survival outcomes, current evidence suggests that RACS achieves comparable, if not superior, OS and DFS rates when measured against conventional laparoscopic techniques (78). The heightened precision afforded by robotic systems facilitates more accurate dissection and resection, potentially enhancing oncological clearance, especially in challenging rectal cancer cases where meticulous pelvic dissection is crucial. Moreover, robotic platforms have demonstrated superior accuracy in performing TME, a procedure essential for reducing local recurrence rates—an important indicator of surgical success (79). The reduction in positive circumferential resection margins (CRM) observed in some robotic-assisted procedures further emphasizes the potential of these technologies to improve oncological outcomes (80).

### 5.2 Functional outcomes

While achieving oncological control is paramount in colorectal cancer management, postoperative functional outcomes are increasingly recognized as essential to optimizing patient quality of life (81, 82). Factors such as postoperative recovery, bowel function, and patient-reported outcomes serve as critical measures of the success of MIS in preserving physiological function while ensuring therapeutic efficacy (83).

Robotic-assisted surgery has been consistently associated with shorter hospital stays, reduced postoperative pain, and a quicker return to routine activities, reflecting its superiority in immediate recovery compared to traditional open surgery (84). These advantages are largely attributable to smaller incisions, reduced intraoperative blood loss, and the enhanced precision of robotic systems, all of which contribute to minimizing surgical trauma. Furthermore, the preservation of long-term bowel function is a crucial consideration. Robotic platforms, with their ability to enable precise nerve-sparing techniques, play a pivotal role in maintaining autonomic nerve integrity, thereby reducing the risk of postoperative complications such as urinary incontinence, fecal incontinence, and sexual dysfunction (72, 85, 86). These considerations are particularly relevant in procedures such as low anterior resection and TME, where preserving pelvic nerve function through accurate mesorectal fascia dissection is critical.

Patient-reported quality of life (QoL) following robotic-assisted colorectal surgery consistently shows improvements across key domains, including physical health, mental well-being, and social functioning (87, 88). The minimally invasive character of robotic procedures, combined with their function-preserving capabilities, results in fewer disruptions to daily life and enhanced long-term satisfaction. Notably, robotic systems enable sphincter-preserving procedures, which markedly improve quality of life by avoiding the need for permanent colostomies and preserving bowel continuity (89).

### 5.3 Comparison of surgical approaches

The evolution of minimally invasive techniques in colorectal surgery has led to the development of various approaches, each with distinct advantages and limitations. Robotic-assisted surgery, laparoscopic surgery, and transanal total mesorectal excision (taTME) are three key methods currently employed in clinical practice (90).

Laparoscopic colorectal surgery has long been established as a standard minimally invasive approach, offering reduced postoperative pain, shorter hospital stays, and quicker recovery compared to open surgery (91). However, laparoscopic techniques are often constrained by limited dexterity and two-dimensional visualization, which can impede precision, particularly in anatomically complex regions such as the pelvis (92). In contrast, robotic-assisted surgery overcomes these limitations by offering enhanced 3D visualization, superior dexterity via wristed instruments, and improved ergonomics for the surgeon (93). These advantages translate into greater precision, particularly in deep pelvic dissections, and potentially superior oncological and functional outcomes.

TaTME is a relatively novel approach that has garnered attention for its ability to achieve high-quality mesorectal excision in patients with mid-to-low rectal cancer (94). The transanal approach offers enhanced visualization of the distal rectum, facilitating more precise dissection and allowing for highly accurate resections. While taTME holds promise, particularly for difficult-to-reach tumors, it also presents challenges, including a steep learning curve and concerns regarding increased recurrence rates in some studies (95, 96). Compared to both laparoscopic and robotic techniques, taTME may offer unique advantages in select cases, but its risk-to-benefit ratio must be carefully evaluated in light of these concerns (97).

In summary, the landscape of minimally invasive colorectal surgery has evolved considerably, with robotic-assisted techniques offering distinct advantages in both oncological and functional outcomes. The comparison of surgical approaches emphasizes the importance of individualized treatment planning, wherein the choice of technique is driven by patient-specific factors, tumor characteristics, and the expertise of the surgical team. As the field continues to advance, ongoing research and refinement of these techniques will further clarify the optimal strategies for achieving both curative and functional success in colorectal cancer surgery.

## 6 Complications and management strategies in robotic-assisted colorectal surgery

### 6.1 Current complications

Despite the numerous advantages offered by RACS, complications remain an inevitable risk, as with any complex surgical intervention. These complications are typically classified into intraoperative and postoperative challenges, each presenting distinct obstacles that may hinder optimal surgical outcomes.

Intraoperatively, the steep learning curve associated with robotic surgery presents a significant challenge, even for seasoned surgeons (98, 99). Issues such as suboptimal port placement, technical malfunctions, and difficulty in navigating intricate anatomical regions can lead to prolonged operative times and increased intraoperative blood loss. Additionally, the dependence on advanced technology introduces the potential for system failures, which may necessitate conversion to open or laparoscopic techniques, thereby diminishing the anticipated benefits of a minimally invasive approach. Pelvic surgery, particularly in the context of rectal cancer, is especially fraught with technical difficulties due to the constrained operative field, heightening the risk of vascular and nerve injury, which can have profound postoperative implications (100).

Postoperatively, patients undergoing RACS are susceptible to a range of complications, some of which are unique to the robotic platform. Anastomotic leaks, one of the most dreaded complications in colorectal surgery, carry serious implications for morbidity and mortality (101). Other postoperative concerns include infections, particularly deep surgical site infections, delayed bowel function recovery (ileus), and thromboembolic events (102). Although robotic techniques have demonstrated a reduction in wound infections and shorter hospitalizations, these benefits do not entirely negate the possibility of postoperative complications, which demand vigilant surveillance and timely intervention to prevent escalation.

### 6.2 Prevention techniques

The prevention of complications in RACS hinges on meticulous preoperative planning and precise intraoperative management (103). Preoperative risk assessment is critical in identifying patients who may be more vulnerable to complications, thus enabling the customization of surgical strategies. This process typically involves comprehensive imaging to assess tumor location and complexity, along with thorough evaluations of patient comorbidities, which can significantly influence intraoperative decisions and postoperative recovery.

Intraoperatively, the prevention of complications is closely linked to the deployment of advanced monitoring technologies and strict adherence to well-established surgical protocols (104). For instance, the integration of intraoperative imaging methods, such as fluorescence-guided surgery, enables real-time visualization of tissue perfusion, facilitating the accurate assessment of anastomotic viability and reducing the likelihood of leaks (105, 106). Furthermore, intraoperative neurophysiological monitoring serves a vital role in safeguarding pelvic nerves during dissection, which is crucial for preserving postoperative urinary and sexual function (107). The precision inherent in robotic platforms enhances the implementation of these preventive strategies, thereby contributing to superior clinical outcomes.

Additionally, strict adherence to standardized protocols—encompassing optimal port placement, efficient robotic docking, and stringent adherence to oncological principles such as ensuring negative circumferential resection margins—can substantially reducing the risk of intraoperative complications (108). Proactive measures, including the use of hemostatic agents and preemptive fluid management strategies, further reduce intraoperative risk and enhance surgical safety (109, 110).

### 6.3 Management protocols

When complications do arise, prompt detection and timely intervention are imperative to limit their impact on patient outcomes. Early identification of postoperative complications, such as anastomotic leaks or infections, requires a high level of clinical vigilance (111). This is often facilitated by the adoption of enhanced recovery after surgery (ERAS) protocols, which emphasize close postoperative monitoring, early mobilization, and active patient participation in their recovery process (112). The use of standardized postoperative care pathways allows for early detection of warning signs, enabling swift diagnostic imaging and laboratory investigations to confirm the presence of complications and guide appropriate therapeutic interventions.

The management of complications in RACS is further optimized through a multidisciplinary approach, in which surgeons collaborate with anesthesiologists, radiologists, and specialized nursing teams to provide comprehensive patient care. For instance, the management of anastomotic leaks may necessitate both surgical and non-surgical interventions, ranging from percutaneous drainage and antibiotic therapy to reoperation, depending on the severity of the complication (113, 114). Similarly, the management of thromboembolic events requires coordinated efforts between the surgical and hematology teams to ensure timely anticoagulation, while carefully balancing the risk of bleeding (115–117). This multidisciplinary approach ensures that complications are addressed holistically, encompassing not only immediate clinical needs but also the long-term implications for patient recovery and quality of life.

## 7 Future directions and new treatment strategies in robotic-assisted colorectal surgery

### 7.1 Emerging therapies

The landscape of chemotherapeutic agents is progressively advancing to selectively target molecular pathways integral to the pathogenesis of colorectal cancer, including those regulating angiogenesis, programmed cell death (apoptosis), and the tumor microenvironment (118, 119). Notably, inhibitors of vascular endothelial growth factor (VEGF) and epidermal growth factor receptor (EGFR) have demonstrated significant efficacy when employed in conjunction with robotic-assisted surgical techniques, particularly in advanced or recurrent cases where conventional chemotherapy alone proves inadequate (120). This synergistic approach augments the precision of tumor excision, offering the potential to enhance both oncological outcomes and overall patient survival (121).

Immunotherapy, particularly immune checkpoint inhibitors targeting programmed cell death protein 1 (PD-1) and cytotoxic T-lymphocyte-associated antigen 4 (CTLA-4), has emerged as a cornerstone of colorectal cancer treatment (122). These inhibitors restore the immune system’s capacity to recognize and eliminate cancer cells, especially in patients with MSI-H tumors, which exhibit heightened sensitivity to immunotherapy (123). The integration of robotic-assisted surgery, facilitating meticulous tumor excision, in conjunction with immunotherapy may reduce recurrence rates and improve long-term survival outcomes. (**Figure 2C**).

### 7.2 Personalized medicine

The advent of personalized medicine has ushered in a paradigm shift in the management of colorectal cancer (124). Advances in genomic sequencing now permit the identification of critical mutations—such as those in KRAS, NRAS, and BRAF—enabling the customization of therapeutic strategies (125). The incorporation of this molecular insight into robotic-assisted surgery allows for highly tailored surgical interventions, informed by the tumor’s unique genetic profile. For instance, patients harboring specific mutations may benefit from more extensive resections, while others may be candidates for minimally invasive procedures that prioritize functional preservation (126).

Pharmacogenomics further complements personalized medicine by elucidating how individual genetic variability modulates responses to therapeutic agents (127, 128). This knowledge enables clinicians to optimize drug dosages and reduce adverse effects, thereby enhancing the efficacy of both chemotherapeutic and immunotherapeutic regimens. Such personalized approaches markedly improve survival outcomes and quality of life by reducing treatment-related toxicity.

### 7.3 Research and clinical trials

Clinical trials remain indispensable in the evolution of robotic-assisted colorectal surgery and treatment paradigms. Ongoing studies are evaluating the combination of robotic surgery with neoadjuvant immunotherapy, investigating whether such regimens can downstage tumors and facilitate less invasive surgical interventions (35, 129). Additionally, research on oligometastatic colorectal cancer is exploring the potential for robotic precision to achieve complete resection of metastatic lesions, potentially prolonging disease-free survival (130).

Further investigation is dedicated to refining perioperative care through ERAS protocols, specifically adapted for robotic-assisted procedures. These efforts are crucial in minimizing complication rates, shortening hospital stays, and accelerating postoperative recovery, thereby improving patient outcomes in the colorectal cancer cohort.

## 8 Conclusion

Robotic-assisted colorectal surgery has emerged as a pivotal innovation in the contemporary management of colorectal cancer, offering unmatched precision and flexibility, particularly in anatomically complex areas such as the lower rectum. Numerous studies consistently affirm the advantages of robotic platforms, highlighting their superior control and enhanced visualization, which significantly enhance the surgeon’s ability to navigate the intricacies of confined pelvic spaces. When compared with conventional laparoscopic techniques, robotic-assisted approaches have demonstrated reduced intraoperative blood loss, shortened hospital stays, and expedited patient recovery, all while maintaining the oncological rigor required to secure clear resection margins and low recurrence rates (131). The corresponding improvements in postoperative quality of life further emphasize the superiority of robotic-assisted methods, especially for complex colorectal cancer surgeries (132).

The efficacy of robotic surgery is further magnified when embedded within a multidisciplinary treatment framework (133). This comprehensive approach, uniting the expertise of oncologists, radiologists, pathologists, and surgeons, ensures that all facets of a patient’s disease are thoroughly addressed. Such a collaborative strategy enhances decision-making, reduces perioperative risks, and contributes to better survival outcomes. This teamwork is particularly indispensable in the management of colorectal cancer, where personalized treatment plans tailored to the patient’s unique disease characteristics can optimize both immediate and long-term outcomes.

To fully harness the benefits of robotic-assisted colorectal surgery, the development and widespread adoption of standardized protocols are crucial. The current variability in surgical techniques and perioperative management across institutions emphasizes the need for universally accepted, evidence-based guidelines (134). Standardizing practices will ensure consistent delivery of high-quality care, reducing discrepancies in outcomes and enhancing patient safety. Moreover, ensuring uniform and comprehensive training in robotic techniques is crucial to ensuring that surgeons across institutions are proficient in the use of these advanced systems (135).

Ongoing innovation and research are critical to further refining robotic surgery. Technological advancements, such as enhanced haptic feedback, machine learning-assisted decision support, and the integration of artificial intelligence, hold tremendous potential to improve the precision and efficiency of robotic procedures. Furthermore, long-term oncological studies are essential to conclusively determine the superiority of robotic-assisted surgery over alternative approaches, particularly concerning survival outcomes, recurrence rates, and sustained quality of life.

Looking to the future, several areas merit focused investigation to strengthen the role of robotics in colorectal cancer surgery. Advanced intraoperative imaging, including fluorescence-based visualization and artificial intelligence-driven data analytics, may enable surgeons to refine resection margins while identifying critical structures in real time, thereby reducing complications and improving oncological control (136, 137). Augmented reality applications promise an additional layer of precision, allowing overlays that highlight blood vessels, nerves, and tumor borders, which can guide more precise dissections (138). As genomic profiling of colorectal cancer continues to elucidate tumor-specific characteristics, integrating these insights with the dexterity of robotic platforms could permit targeted resections that maintain organ function. Simultaneously, combining robotic surgery with cutting-edge immunotherapies—such as checkpoint inhibitors—and highly selective chemotherapeutic agents has the potential to address residual disease more effectively, shortening recovery times and prolonging survival. Finally, the establishment of uniform training curricula and certification standards across institutions will help ensure equitable access to these technologies and uphold consistently high levels of surgical care.

## Glossary

CRC: Colorectal Cancer
RACS: robotic-assisted colorectal surgery
TME: total mesorectal excision
TNT: total neoadjuvant therapy
dMMR: mismatch repair deficiency
MSI-H: high microsatellite instability
MIS: minimally invasive surgery
RAS: robot-assisted surgery
3D: three-dimensional
LARC: locally advanced rectal cancer
pCR: pathological complete response
DFS: disease-free survival
OS: overall survival
ICIs: immune checkpoint inhibitors
MSS: microsatellite-stable
AR: augmented reality
CME: continued medical education
ISR: intersphincteric resection
CRM: circumferential resection margins
QoL: quality of life
TaTME: transanal total mesorectal excision
ERAS: enhanced recovery after surgery
VEGF: vascular endothelial growth factor
EGFR: epidermal growth factor receptor
PD-1: programmed cell death protein 1
CTLA-4: cytotoxic T-lymphocyte-associated antigen 4
